# Obituary Dr. David Sutherland MD, PhD Emeritus Professor of Surgery, University of Minnesota 25th December 1940–23rd March 2025

**DOI:** 10.3389/ti.2025.14693

**Published:** 2025-04-10

**Authors:** Vassilios Papalois

**Affiliations:** Department of Surgery, Imperial College London, London, United Kingdom

**Keywords:** David Sutherland, pancreas transplantation, islet transplantation, diabetes, chronic pancreatitis

The passing of Dr David Sutherland on the 23rd of March 2025 (Figure 1), deeply saddened the ESOT transplant community. I was truly humbled to receive the gracious invitation of the ESOT Executive to write this obituary on behalf of our Society, expressing our deepest respect and sorrow for the loss of one of the most distinguished servants of our vocation whose passing signified the end of a great transplant era.

**FIGURE 1:**
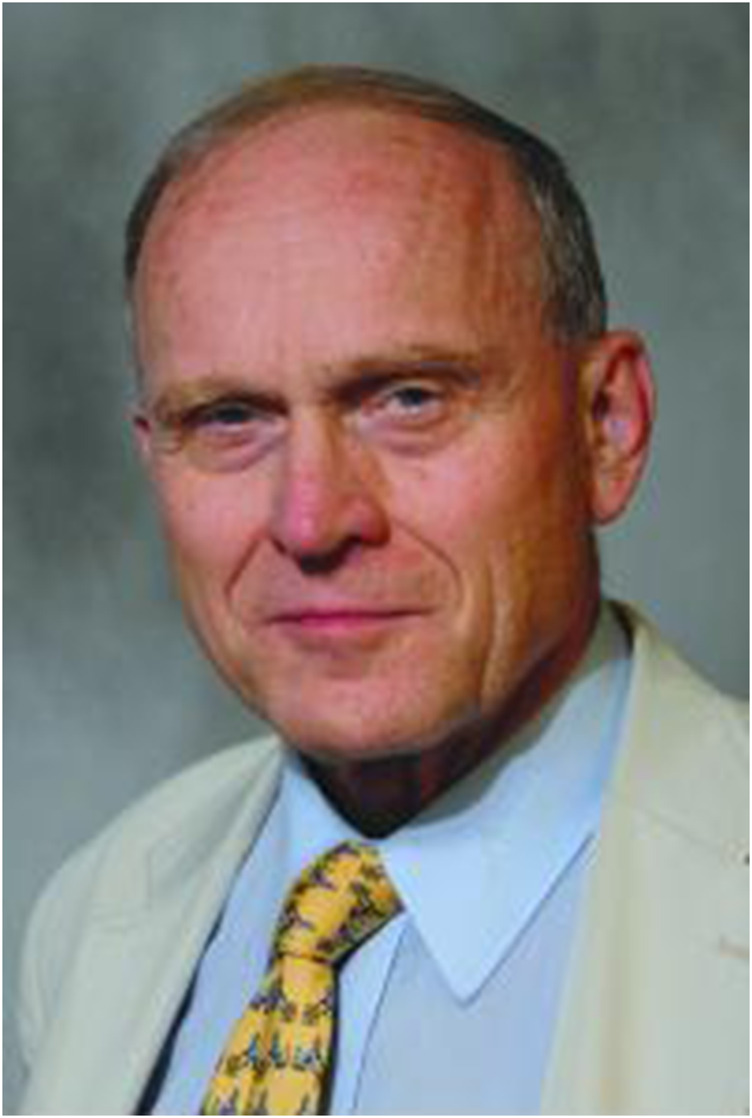
Dr David E.R. Sutherland, 1940–2025.

When many colleagues around the world received by Dr. Rainer Gruessner the saddest news that our beloved teacher, mentor and friend Dr. David Sutherland passed away, within a matter of seconds, messages of sincere admiration and respect started pouring from all over the world praising the character, ethos, clinical and research achievements of David Sutherland, a truly remarkable man.

Dr. David Sutherland was a towering figure in the fields of transplant surgery and immunology. His astonishing career spanned nearly six decades, during which he made groundbreaking contributions to the treatment of diabetes, chronic pancreatitis, and organ transplantation.

Dr. Sutherland’s journey began in the early 1960s when he entered the University of Minnesota Medical School, where he graduated in 1966. Even as a medical student, he demonstrated a keen interest in immunology - a field that would define much of his career.

After completing his medical degree, Dr. Sutherland served 2 years in the Army, a period that helped shape his strong sense of discipline, multitasking and leadership. In 1975, he completed his general surgery residency at the University of Minnesota and followed this with a transplant fellowship in 1976. By 1977, he had earned a Ph.D., marking the beginning of a prolific academic and clinical career that would lead him to become one of the foremost figures in the world of transplantation.

Dr. Sutherland joined the faculty at the University of Minnesota, where he would leave an indelible mark on the field of transplantation. He became Professor of Surgery in 1984, and he would go on to direct the University’s prestigious Diabetes Institute for Immunology and Transplantation, a position he held from 1994 for almost three decades. Dr Sutherland was Head of the Division of Transplantation from 1995 to 2009 and was holder of a Diabetes Research Chair since 2003. He was a mentor to countless medical students, surgeons, and researchers, shaping the next-generation of transplant surgeons and scientists. His commitment to education and mentorship was one of his defining qualities. Over 100 transplant surgeons who trained under his guidance went on to lead pancreas and islet transplant programs around the world.

Dr. Sutherland’s academic achievements were equally impressive. He authored over 1500 scientific publications, covering a broad range of topics with particular emphasis on beta-cell replacement therapy for diabetes mellitus, chronic pancreatitis, immunosuppression management, and the training of scientists and clinicians in translational research. He was one of the most cited authors in the history of the field of transplantation.

His work on pancreas and islet transplantation was groundbreaking. In 1974, he conducted the world’s first clinical islet transplant at the University of Minnesota. Dr. Sutherland became the Director of the University of Minnesota’s pancreas transplant program in 1978, making it the oldest and largest pancreas transplant program in the world - with more than 2,400 pancreas and 1,000 islet transplants performed under his leadership. He also performed the world’s first living-donor partial pancreas transplant in 1979. Furthermore, he established a very successful programme of pancreatectomy followed by islet auto-transplantation for the treatment of chronic pancreatitis. In 1980, he founded the International Pancreas Transplant Registry which remains a key resource for clinicians and researchers in the field.

Dr. Sutherland’s influence extended far beyond his own research and clinical work. He was a beloved mentor and leader in the transplant community, holding prestigious leadership roles throughout his career. He served as President of several key organisations, including the American Society of Transplant Surgeons (1990–1991), the Cell Transplant Society (1995–1996), the International Society for Pancreas and Islet Transplantation (1996–1997), and The Transplantation Society (2002–2004).

Dr. Sutherland’s contributions were widely recognised through numerous awards and honours including the Medawar Prize in 2012, the world’s highest dedicated award for the most outstanding contributions in the field of transplantation.

Beyond his astonishing achievements, David Sutherland’s intelligence, work ethic and sense of humour were legendary.

I will share some of my personal experience in working with David Sutherland and I know that it reflects similar stories by many other colleagues.

I met Dr Sutherland for the first time in August of 1988 when I was an elective medical student at the University of Minnesota. I asked his secretary for an appointment, and she told me that “Dr Sutherland will see you at 3 o’clock outside the operating rooms”. I was absolutely certain that this was 3 o’clock in the afternoon but it was actually 3 o’clock in the morning! I still remember that he had three kidney-pancreas transplants going on, there was a parade of fellows waiting for him to review their abstracts, papers, and grant applications and he was also making many phone calls all over the world advising colleagues about difficult patients. A typical David Sutherland day!

Another great memory were the Friday afternoon laboratory meetings. His brain was literally a library, and he could give you all the information you needed about any published paper in the field of transplantation along with a superb critical analysis as to what you can learn from that and apply it in your research. He was challenging the research fellows by putting on the table the most provocative arguments, like “acute cellular rejection does not exist, debate me!” With his knowledge and intelligence, he could almost convince you that acute cellular rejection did not exist! However, the point of this exercise was to push to the limits our thinking and from that, all the great research ideas were coming up! Only David Sutherland could drive such a discussion!

In the clinical setting, David Sutherland has always been pushing the boundaries. I never remember him saying “This we will not do because it is difficult”. If it was difficult, it was our job to do it! What was also most impressive was his amazing ability to critically analyse clinical challenges and generate the right research questions for translational research that was his great research passion. “Do not look in the libraries for ideas, look in front of you, the patients give you the ideas!” he used to say.

It was also amazing to see how the skills of David Sutherland were complementing those of John Najarian, the other transplant giant of the University of Minnesota and David Sutherland’s, mentor. Between the two of them, they created one of the most successful clinical, research and training transplant programmes the world has ever seen.

Beyond the glamour of clinical and academic achievements, David Sutherland was a warm and kind-hearted human being, with a great sense of humour who was utterly loyal to his patients, colleagues and friends. He donated his kidney to his beloved wife Vanessa, and this was just one example of his genuine love and commitment to those who suffer.

Shakespeare wrote in the “Twelfth Night”: “Some are born great, some achieve greatness, and some have greatness thrust upon them”. David Sutherland was one of those unique human beings that had all three: he was born with great talents, he achieved great things, and he also responded to the thrust of life’s challenges with creative action.

In 1989, the “Flame of Hope” was lit in London Ontario in Canada to honour those who discovered insulin and all the people who have lost their lives to diabetes. The flame will remain lit until there is a cure for diabetes. When a cure is found, the flame will be extinguished by the researchers who discover the cure. Whenever David Sutherland was asked if he believed that the flame will ever be extinguished, he was always silent, full of emotion and his eyes were wet but full of light and hope. It is our duty to keep this hope alive until the dream is realised.

Now David Sutherland belongs to the Pantheon of transplant giants. Those of us who had the great fortune to be taught and mentored by him have the duty to pass the torch to the generations that follow. As Henry Adams said: “A teacher affects eternity. He can never tell where his influence stops.”

For more than four decades, ESOT spearheaded within the European and the international transplant community the same causes and values that David Sutherland served with such distinction: commitment to patients, clinical innovation, progressive research, transformative education, equitable-diverse-inclusive spirit. Our Society will treasure and further advance this legacy in honour of David Sutherland and all the legendary figures of transplantation.

David Sutherland, 1940–1925, May He Rest in Peace.

## Data Availability

The original contributions presented in the study are included in the article/supplementary material, further inquiries can be directed to the corresponding author/s.

